# How Useful Is Electroencephalography in the Diagnosis of Autism Spectrum Disorders and the Delineation of Subtypes: A Systematic Review

**DOI:** 10.3389/fpsyt.2017.00121

**Published:** 2017-07-12

**Authors:** Oana Gurau, William J. Bosl, Charles R. Newton

**Affiliations:** ^1^Department of Psychiatry, University of Oxford, Oxford, United Kingdom; ^2^School of Nursing and Health Professions, University of San Francisco, San Francisco, CA, United States; ^3^Benioff UCSF Children’s Hospital Oakland Research Institute, Oakland, CA, United States; ^4^KEMRI-Wellcome Trust Research Program, Centre for Geographic Medicine Research (Coast), Kilifi, Kenya

**Keywords:** autism spectrum disorders, autism, electroencephalography, functional connectivity, spectral analysis, information dynamics

## Abstract

Autism spectrum disorders (ASD) are thought to be associated with abnormal neural connectivity. Presently, neural connectivity is a theoretical construct that cannot be easily measured. Research in network science and time series analysis suggests that neural network structure, a marker of neural activity, can be measured with electroencephalography (EEG). EEG can be quantified by different methods of analysis to potentially detect brain abnormalities. The aim of this review is to examine evidence for the utility of three methods of EEG signal analysis in the ASD diagnosis and subtype delineation. We conducted a review of literature in which 40 studies were identified and classified according to the principal method of EEG analysis in three categories: functional connectivity analysis, spectral power analysis, and information dynamics. All studies identified significant differences between ASD patients and non-ASD subjects. However, due to high heterogeneity in the results, generalizations could not be inferred and none of the methods alone are currently useful as a new diagnostic tool. The lack of studies prevented the analysis of these methods as tools for ASD subtypes delineation. These results confirm EEG abnormalities in ASD, but as yet not sufficient to help in the diagnosis. Future research with larger samples and more robust study designs could allow for higher sensitivity and consistency in characterizing ASD, paving the way for developing new means of diagnosis.

## Introduction

### History and Definition of Autism Spectrum Disorders (ASD) and Its Subtypes

Autism spectrum disorders are a group of lifelong neurodevelopmental disorders. Recent epidemiological research estimates the prevalence of ASD at around 1 in 100 children in the UK (1) and 1 in 68 children in the USA (2). ASD include the following subtypes: autistic disorder, Asperger syndrome, childhood disintegrative disorder, and pervasive developmental disorder-not otherwise specified (PDD-NOS) (3). In a more recent classification in the Diagnostic and Statistical Manual of Mental Disorders, Fifth Edition, all these subcategories are subsumed under “Autism Spectrum Disorder” (4).

Autism spectrum disorder was first described by Kanner in 1943 who identified the triad of core characteristics: impaired social interaction and communication involving reduced eye contact, facial expression, and body gestures, a restricted range of interests and repetitive behavior (5). Research indicates that ASD are on a broad continuum of severity and differences in symptoms can be detected, with the clinical symptoms becoming evident from the second year of life. These features are thought to be the result of atypical neural connections within the brain (6–11). Electroencephalography (EEG) can measure neural activity and may provide a useful tool to detect children at risk of developing ASD and, thus, provide an opportunity for early intervention. In addition, it may help delineate between the subtypes.

Autism may be described as a dynamical disorder and analyzed from the perspective of complex dynamical systems (10, 12–15). Measureable changes in cortical excitability may contribute to, or be a manifestation of, connectivity abnormalities (16). The two concepts, neural connectivity and neural dynamics, are related. For example, studies of complex networks reveal that they can exhibit a kind of “spatial chaos” in which network properties can change drastically with small changes to key network connections, analogous to the sensitive dependence of chaotic time series on initial conditions (17). Therefore, computing of dynamical system features of the brain from EEG time series may be used to infer atypical neural connectivity that is associated with autism. Although neural connectivity can be measured directly using diffusion tensor imaging, non-linear time series analysis methods have begun to provide a tool for detecting differences in neural connectivity measured with EEG devices on multiple smaller scales through quantitative analysis of signal complexity (12, 18–21).

The interpretation of the EEG may be complicated by the presence of epilepsy, which develops in adolescence in one-third of the patients. Subtypes of ASD include Asperger syndrome, which involves social symptoms, with typical language development and non-verbal intelligence. PDD-NOS differs from autistic disorder by lacking repetitive behaviors or evident social deficits. Disintegrative Disorder is a severe form of autism acquired after normal development until 2–10 years of age. This phenotypic diversity in ASD also involves a varying degree of impairment in each symptom category between individuals (22). These are not the only subtypes of ASD. But these are the most encountered ones in research papers, particularly the ones selected for this review.

The implication for neural connectivity disorders such as autism is that EEG analysis may reveal neural network abnormalities that are related to functional and behavioral symptoms associated with the disorder. Reliable and relatively low cost, simple EEG measurements may provide important clinical biomarkers for early risk assessment and for monitoring the condition’s progression. The aim of this review is to evaluate evidence for the utility of EEG in identifying such abnormal activity for the diagnosis of ASD and for the delineation of its subtypes.

### Electroencephalography (EEG) and Quantitative EEG (qEEG)

Scalp EEG sensors measure the summed potentials of several millions of neurons. The physiological interpretations of the recorded signal describe both intrinsic properties of the neurons such as their ionic conductance, as well as connectivity characteristics and neural networks interactions (23). These characteristics are typically classified in five “classic” frequency bands: delta (0–4 Hz), theta (4–8 Hz), alpha (8–12 Hz), beta (12–30 Hz), and gamma (30–100 Hz), but the definitions of these bands may vary between studies. These frequencies characterize different states of the brain, each with a specific function, physiology, and cortical topography. However, newer methods of EEG analysis, such as using multiscale entropy (12, 13, 24), measure “scales” rather than the traditional frequency bands.

Evaluation of the power of EEG signals in various frequency bands and the nature of connectivity between brain regions using correlation analysis is commonly performed using qEEG: a collection of computerized tools and algorithms to analyze the EEG signal (25). The qEEG encompasses methods of EEG analysis, such as spectral analysis, functional connectivity analysis, and, recently, information dynamics, and is used in the search for quantitative features associated with altered behaviors in ASD. The values computed using various methods of qEEG analysis may be collectively referred to as EEG signal features.

The specific questions that we set out to address in this review were as follows: (i) can analysis of EEG be used to detect subjects with ASD, in particular is it useful in the diagnosis and (ii) can EEG features identify subtypes of ASD?

## Methods

### Search and Selection Strategy

The literature search was conducted in three peer-reviewed databases: PubMed, Embase, and PsycInfo. Keyword searches were performed in order to identify the most suitable studies for this review. The key terms used were “ASD,” “Asperger,” “autism,” “EEG,” “encephalography,” “spectral analysis,” “functional connectivity,” and “information dynamics”. On each database, the searches consisted of each of the three key terms describing the disorder and subtypes plus each of the terms describing the methods (Table 1).

**Table 1:** Description of the search strategy.

Search element	PubMed	Embase	PsycInfo
Disorder	Autism spectrum disorders (ASD)	ASD	ASD
	Autism	Autism	Autism
	Asperger	Asperger	Asperger
Method	Electroencephalography (EEG)	EEG	EEG
	Encephalography	Encephalography	Encephalography
	Spectral analysis	Spectral analysis	Spectral analysis
	Functional connectivity	Functional connectivity	Functional connectivity
	Information dynamics	Information dynamics	Information dynamics

In addition, to select the studies to be reviewed, the following inclusion criteria were used:

1. The studies were performed on humans, either children or adults;
2. A comparison was performed between either: (i) ASD patients and healthy controls or (ii) ASD patient with different subtypes;
3. The studies’ outcome consisted of specific EEG features of ASD, not its comorbidities;
4. The studies included patients diagnosed according to the clinical criteria from DSM III onward;
5. The studies were published between 1980 and May 2016;
6. The studies were performed using EEG and the signal was analyzed using spectral analysis, functional connectivity measures, or information dynamics methods.

**Figure 1:**
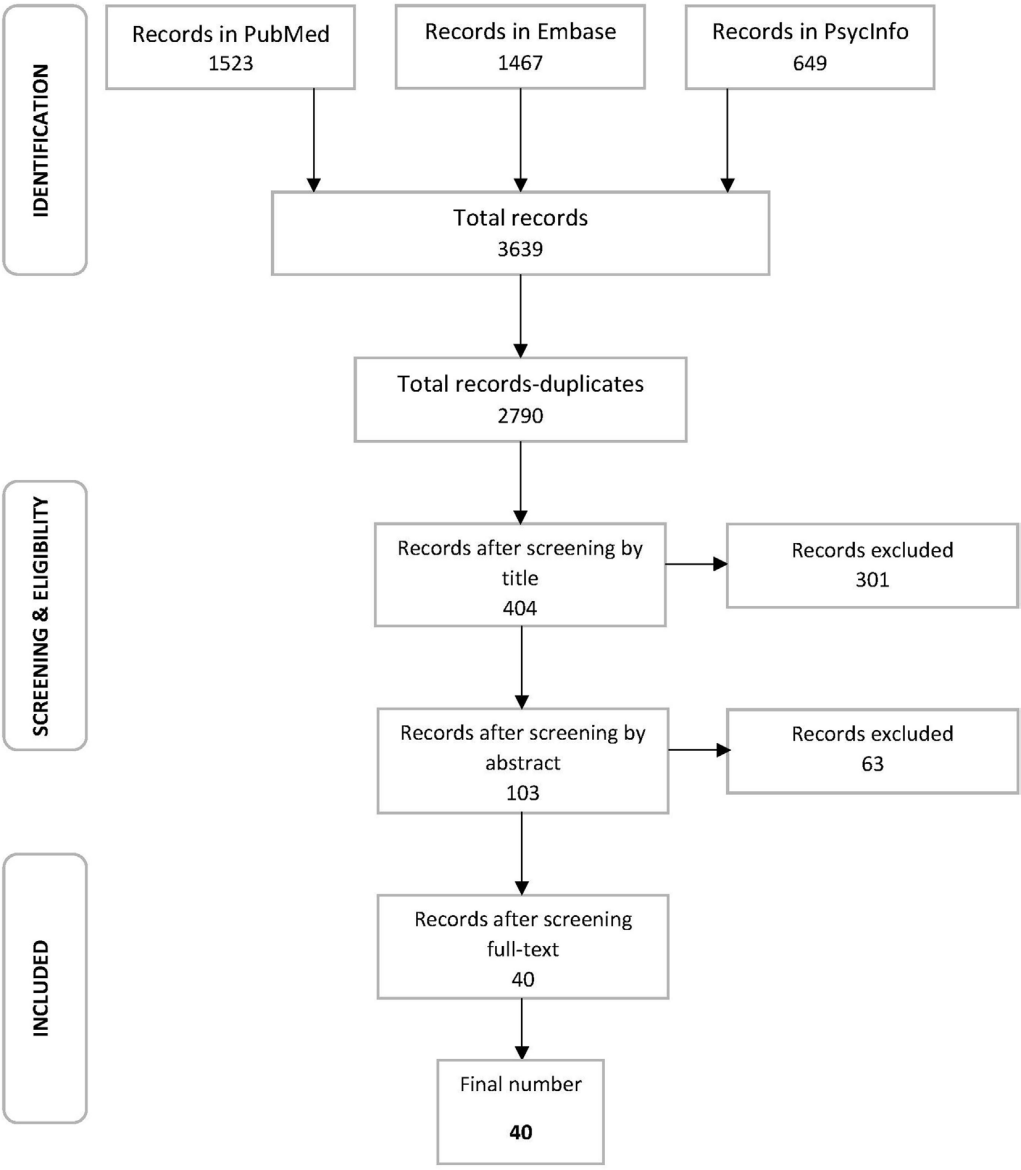
Flow diagram presenting the process of study selection, including the three-step strategy used to reach the final collection of studies and number of records in every step.

In order to filter the initial number of titles obtained (Table 2), according to the six eligibility criteria, a three-step strategy was followed (Figure 1). First, the titles of each of the papers yielded by the initial search were examined for relevance to the topic. The absence of any of the key words from the titles led to the exclusion of the studies. Second, the abstracts were scanned for relevance, as they briefly indicate the methods used in the study and their results. Animal studies, review papers, or studies combining different methods of analysis were excluded. The review papers were used as sources for relevant papers to be examined. Lastly, a full text analysis was performed, allowing a closer examination of diagnostic criteria of the patients and the quality of their results. This process yielded the final collection of studies used for data extraction in the review (Figure 1).

**Table 2:** Total number of papers identified from each database before and after the exclusion of duplicates.

Database	Total number of titles	Total number of titles-duplicates
PubMed	2,155	1,523
Embase	2,095	1,467
PsycInfo	964	649

Following the selection stage, the relevant data were extracted from each paper. For this purpose, in consistency with the PRISMA 2009 checklist (http://www.prisma-statement.org/), a template with a number of descriptive variables for each study such as its authors and publishing year; a detailed section describing the methods used in the study, including description of the patient and the control groups, the task performed, methods of EEG analysis as well as statistical tests; and a summary of the results was used. For consistency, all data were extracted from the studies using this template.

Inspection of the literature selected for data extraction revealed three types of EEG signal analysis used for detection of ASD: (i) spectral analysis; (ii) functional connectivity and coherence analysis; and (iii) a larger category, information dynamics, including several methods based on dynamical systems theory and mathematical concepts.

### The Methods Categories

Functional connectivity evaluates the relationship between the signals recorded at different brain regions simultaneously. Usually these relationships are quantified in terms of some measure of synchronization between two signals. Synchronization may be defined in several different ways (26, 27). Variations of this analysis include coherence, phase locking index, phase synchronization, phase lag index, and synchronization index. Functional connectivity analysis also involves measures of embedded data, such as cross recurrence diagrams and synchronization likelihood. Often signals are decomposed into standard frequency bands.

Spectral analysis is the most common quantitative method used for EEG signal analysis and interpretation. It breaks the continuous range of frequencies into defined bands and evaluates the signal distribution over several frequency bands, usually as the five divisions described above. The spectral power may be computed for each frequency band at each sensor (28). Sometimes the total power in each frequency band is summed over all sensors, giving a single power value over the entire scalp for each frequency band. For example, a power value for the alpha band over the entire scalp may be reported. Or, power in the alpha band at each sensor location may be reported. Group differences between populations with autism and typically developing controls were assessed. Results were presented either as absolute power or relative power (the ratio of band power to total power over bands).

Information dynamics methods make use of non-linear analysis methods. These include various measures of entropy, or other dynamical concepts such as Lyapunov exponents or recurrence plot analysis, among others. The meaning of these concepts has been derived from physical systems, but it is not clear how they related to neural systems. Thus, significant correlations between non-linear features and neural or behavioral observations are sought using machine learning algorithms and mathematical classifiers based on neural networks, graph theory, fractals, or Bayesian methods. Machine learning algorithms and classifiers use a set of rules characterizing members of one or more categories and then apply these rules to a dataset for classification. The most commonly used non-linear feature used in neuroscience has been multiscale entropy, a measure of signal complexity. The original EEG signal is used to create a sequence of coarse-grained time series. Each scale of entropy is obtained as follows: scale 2 is calculated by averaging every two values, scale 3 time series is obtained by averaging every three values, etc. The sample entropy is then computed for each time series to produce entropy as a function of time. This coarse graining procedure was first introduced to signal analysis by Costa et al. (29). Although this procedure is commonly used, it has only recently been noted that for powers of 2 (1, 2, 4, 8, …) the coarse graining procedure is mathematically identical to the Haar wavelet transform (30). Much is known about wavelet transforms and their relationship to frequency decomposition (31). Multiscale entropy is, thus, a computation of sample entropy on each of the wavelet transform scales.

According to the PRISMA 2009 checklist, the analysis of the utility of each of these methods includes a general description, a critical evaluation of statistically significant evidence to differentiate between ASD patients and non-ASD subjects, or different ASD subtypes extracted from the literature, advantages the technique holds over others, as well as gaps and future lines of improvement followed by a conclusion summarizing the overall prospects of using it as a tool in ASD detection.

## Results

An evaluation of the selected literature led to the presentation of the papers in three EEG signal analysis methods used to characterize ASD described above.

### Functional Connectivity

Of the 40 studies selected for review, 12 studies compared functional connectivity between ASD patients and non-ASD subjects. All studies reported at least one statistically significant difference in ASD connectivity in at least one frequency band. Of the 12 studies employing this method of analysis, 10 use coherence as a measure of connectivity, 1 calculated the phase lag index of the time series, and 1 calculated clustering to determine the level of synchronization. EEG recordings were performed under relaxed, no task conditions, with eyes either open or closed, during sleep or during an object recognition, audio or video task. The overall patterns in results are presented in Table 3 together with the condition of the EEG recording and the principal measure of each study.

**Table 3:** Studies using functional connectivity.

Paper	Patients characteristics	Controls characteristics	Condition	Measure	Changes in ASD
Orekhova et al. (32, 33)	*n* = 28; mean age = 14.4 months; sex = 18 F, 10 M; HR	*n* = 10; mean age = 38 months; sex = 3 F, 7 M; High-risk ASD (HR-ASD)	3 video stimuli	De-biased weighted phase lag index	HR-ASD: hyper-connectivity in alpha band in frontal and central areas (*p* < 0.05)
	*n* = 26; mean age = 14.7 months; sex = 14 F, 12 M; Low-risk (LR)	*n* = 18; mean age = 38 months; sex = 15 F, 3 M; High-risk non-ASD			The degree of hyper-connectivity correlated with the severity of ASD symptoms in later diagnosed

Righi et al. (34)	*n* = not specified; adults HR of ASD Had an older sibling with ASD	*n* = not specified; adults LR of ASD	Speech sounds	Coherence	Infants at risk: reduced connectivity (*p* < 0.005)
					Connectivity: HR-ASD < HR-NASD < LR

Barttfeld et al. (35)	*n* = 10; mean age = 23.8 years; sex = 1 F, 9 M; subtypes = autism, Asperger syndrome	*n* = 10; mean age = 25.3 years; sex = 1 F, 9 M	Relaxed eyes closed	Coherence (synchronization likelihood)	Delta band: decreased long-range connectivity (fronto-occipital) and increased short-range connectivity (frontal lateral) in ASD (*p* < 0.05)

Murias et al. (36)	*n* = 18; adults sex = 18 M diagnosis = ASD; Some were taking medication	*n* = 18; age-matched sex = 18 M	Relaxed eyes closed	Coherence	Theta: increased connectivity in frontal and temporal left hemisphere (*p* < 0.025)
					Alpha: decreased long-range connections of the frontal area (*p* < 0.025)

Leveille et al. (37)	*n* = 9; mean age = 21.1 years; diagnosis = ASD	*n* = 13; mean age = 21.5 years	Rapid eye movement (REM) sleep	Coherence	Theta and delta: increased long-range coherence between the occipital region and the rest of the brain and decreased the frontal area (*p* < 0.05)

Boersma et al. (38)	*n* = 12; mean age = 3.5 years; sex = 2 F, 10 M; subtypes = autism (2), Asperger syndrome (1), PDD-NOS (9); average IQ = 85	*n* = 19; mean age = 3.5 years; sex = 19 M; average IQ = 108	Pictures of cars	Clustering	Overall whole brain under-connectivity in beta, theta, and alpha bands (*p* < 0.01)

Catarino et al. (13, 39)	*n* = 15; mean age = 31 months; diagnosis = ASD	*n* = 15; mean age = 29 months	Object recognition	Coherence	Decreased coherence for both tasks in alpha and theta bands (*p* < 0.05)

Carson et al. (40)	*n* = 19; mean age = 9.9 years; sex = 1 F, 19 M; diagnosis = ASD	*n* = 13; mean age = 10 years; sex = 4 F, 9 M	Videos of someone reading a story	Coherence	Decreased coherence in alpha band in frontal and temporal lobes at baseline (*p* < 0.05)

Cantor et al. (41)	*n* = 11; age range = 4–12 years; sex = 2 F, 9 M; subtypes = autism	*n* = 119; classification = normal children (*n* = 88, age range = 5–15 years), a matched group of intellectually disabled children (*n* = 18, age range = 5–15 years) and a group of mentally age-matched normal toddlers (*n* = 13, age range = 16 months to 5 years)	Relaxed eyes open	Coherence	Higher coherence between and within hemispheres in delta and alpha bands (*p* < 0.05)

Chan et al. (42, 43)	*n* = 21; mean age = 10.27 years; sex = 2 F, 19 M	*n* = 21; mean age = 9.85 years; sex = 7 F, 14 M	Object recognition	Coherence	Increased frontal coherence in left hemisphere in theta bands (*p* < 0.05)

Coben et al. (44)	*n* = 20; mean age = 6–11 years; sex = 6 F, 14 M	*n* = 20; mean age = 6–11 years; sex = 6 F, 14 M	Relaxed eyes close	Coherence	Decreased coherence in theta and delta bands in frontal region (*p* < 0.005), delta, theta, and alpha in temporal region (*p* < 0.05) and delta, theta, and beta in parietal and occipital regions (*p* < 0.05)

Buckley et al. (45)	*n* = 87; age range = 2–6 years; diagnostic = ASD	*n* = 29; age range = 2–6 years (TYP)	Awake, slow-wave sleep, and REM sleep	Coherence, phase lag,	Increased coherence observed in ASD compared to TYP, almost exclusively during slow-wave sleep, in the frontal–parietal areas, in long-distance pairs
	*n* = 21; age range = 2–6 years; diagnosis = developmental delay without ASD (DD)				

Lazarev et al. (46)	*n* = 14; age range = 6–14 years; sex = 14 M; diagnosis = ASD	*n* = 19; age range = 6–14 years; sex = 19 M	Intermittent photic stimulation	Coherence	Significantly lower coherence in ASD than the control group in the beta frequencies

*n, number of subjects; F, female; M, male; PDD-NOS, pervasive developmental disorder-not otherwise specified; REM, rapid eye movement; ASD, autism spectrum disorders; HR-ASD, High-risk ASD; HR, high-risk*.

Although the results are variable, some generalizations can be inferred. Thus, ASD is usually associated with reduced long-range connections in the alpha band between the frontal lobe and other brain regions. This finding supports the under-connectivity theory of ASD, which is supported by fMRI studies (47). Seven out of 11 studies performed coherence analyses in the alpha band, of which 4 supported the presence of under connectivity. For instance, Murias et al. studied coherence in ASD in a relaxed eyes closed condition, using a high-density EEG montage (124 electrodes) in male adults and found significant differences in the alpha band between patients and controls, with reduced long-range connections particularly from the frontal areas. Some of the patients were taking medication, which may have influenced the results (36). Catarino et al. also identified an overall decrease in brain connectivity in the alpha band, in subjects with ASD performing two object recognition tasks (39). In a more recent study, Carson et al. performed a study on a younger pool of participants that replicated the decrease in the long-range connectivity in the alpha band, while they were attending to a video of either a familiar or unfamiliar person readying a story (40). The fourth study to support this theory calculates clustering as a measure of brain connectivity. Boersma et al. used graph analytical tools to demonstrate reduced whole brain connectivity in toddlers, particularly in the alpha band (38). However, three of the six studies show contradictory results. Orekhova et al. performed a longitudinal study, testing participants at high risk (HR) and low risk (LR) of developing ASD at 14 months and at 38 months, after part of the HR participants were diagnosed with ASD. The study showed significant differences between the children diagnosed with ASD and the other participants, with hyper-connectivity in the alpha band between the frontal and central areas in those at risk of developing autism (33). Testing participants in a relaxed eyes open condition, Cantor et al. supports the decrease in alpha connectivity, this time within and between hemispheres (41). Buckley et al. tested participants aged 2–6 years old during three sleep state conditions and found an increase in coherence in ASD patients compared to neurotypical subjects, particularly in long-range connections in the frontal–parietal areas (45).

Anatomical and functional studies have also demonstrated short-range, local over connectivity, reflected in increases of short-range association fibers (48). Moreover, it is known that theta oscillations underlie locally dominant processes (49). Six out of 11 studies investigated connectivity differences in this frequency band: 3 of the studies supporting previous fMRI findings (36, 37, 43) and 3 not finding any evidence of over connectivity (38, 39, 44). Murias et al. identified significant over connectivity in the theta band in the frontal and temporal areas of the left hemisphere (36). Leville et al. studied participants during rapid eye movement sleep and identified increased long-range connectivity between the visual area V1 in the occipital lobe and other parts of the brain (37). During an object recognition task, Chan found increased frontal coherence in the theta band, confirming fMRI findings (43); but three studies found whole brain under connectivity in the theta band, the first two employing object recognition tasks (38, 39), while Coben et al. demonstrated under connectivity in the frontal, temporal, and parietal regions (44).

The delta and beta bands yielded significant results in 6 of the 11 functional connectivity studies, but lacked consistency. Barttfeld et al. showed ambivalent results in the delta band, with decreased long-range delta connectivity between the frontal and occipital areas and increased short-range delta connectivity in the frontal region (35). In a sleep study, Leville et al. had contradictory results, showing increased long-range delta connectivity between the occipital area and the rest of the brain (37). Coben also shows decreased frontal and temporal coherence in the theta band (44). Decreases in connectivity were detected by Boersma et al. (38), Coben et al. (44), and Lazarev et al. (46) in the analysis of the beta band. Despite these results, it is notable that 9 of the 11 papers did not find any significant difference in the beta band. Generalization, in this case, could not be inferred because of the different conditions of the EEG recordings and the age differences between the participants of each study.

### Spectral Analysis

Twenty-one of the selected studies used spectral analysis to characterize ASD. All studies recorded statistically significant variants in spectral power in ASD patients compared to non-ASD subjects in at least one frequency band. The studies measured differences in relative or absolute spectral power across frequency band, power spectrum density, or spectral properties such as amplitude. The signal was recorded during relaxed conditions with eyes either open or closed, during sleep, cognitive tasks, or while attending to video or audio stimuli. The overall trend of results can be visualized in Table 4, along with participants’ characteristics, recording condition and the main measure of the study.

**Table 4:** Studies using spectral analysis.

Paper	Patients characteristics	Controls characteristics	Condition	Measure	Changes in ASD
Matlis et al. (50)	*n* = 27; mean age = 4–8 years; sex = 2 F, 25 M; subtypes = autism	*n* = 55; mean age = 4–8 years; sex = 26 F, 29 M	Relaxed eyes open	Spectral properties	Reduced posterior/anterior power ratio in the alpha frequency range (8–14 Hz) (*p* ≤ 0.0025)

Sheikhani et al. (51)	*n* = 15; age range = 6–11 years; sex = 5 F, 10 M; subtypes = Asperger syndrome; verbal IQ > 85; handedness = 1 LH, 14RH	*n* = 11; age range = 6–11 years; sex = 4 F, 7 M; handedness = 1LH, 1AD, 9RH	Eyes closed, relaxed eyes opened, looking at 3 puzzle shapes, looking at mother’s and stranger’s pictures upright and inverted	Spectral power	Higher power in gamma band while resting with eyes open (*p* < 0.05)

Cantor et al. (41)	*n* = 11; age range = 4–12 years; sex = 2 F, 9 M; mean IQ = 37.5; subtypes = autism	*n* = 119; classification = normal children (*n* = 88, age range = 5–15 years), age-matched group of mentally disabled children (*n* = 18, age range = 5–15 years) and a group of mentally age-matched normal toddlers (*n* = 13, age range = 16 months to 5 years)	Relaxed eyes opened	relative power total power	ANCOVAs and *t*-tests: Lower alpha power in all regions in ASD subjects compared to age-matched normal and age-matched mentally disabled children (*p* < 0.001)
					Higher power than the normal or mentally handicapped children in the bilateral fronto-temporal and left temporal regions (*p* < 0.005). Lower power in the bilateral occipital regions normal (*p* < 0.05). Lower power than toddlers in the left central, midline central, and left fronto-temporal regions (*p* < 0.05)

Chan et al. (42, 43)	*n* = 17; mean age = 7.1 years; sex = 3 F, 14 M	*n* = 105; mean age = 7.7 years; sex = 61 F, 44 M	Relaxed eyes opened	Spectral profiles: absolute delta, theta, alpha, sensorimotor rhythm, beta; relative delta, theta, alpha, sensorimotor rhythm, beta bands	Absolute amplitudes: higher amplitudes in all five frequency bands (*p* < 0.005)

Chan et al. (42, 43)	*n* = 66; age range = 5–18 years; sex = 6 F, 60 M	*n* = 90; age range = 6–12 years; sex = 42 F, 48 M	Relaxed eyes opened	Mean absolute and relative power of typically developing children and children with ASD	ASD less relative alpha (91% sensitivity, 73% specificity) and more relative delta (76% sensitivity, 78% specificity)

Daoust et al. (52)	*n* = 9; age range = 12–53 years; sex = 1 F, 8 M; diagnosis = ASD; subtypes = autism, Asperger syndrome	*n* = 8; age range = 8–56 years; sex = 1 F, 7 M	Relaxed, eyes closed morning and evening and sleep	Awake absolute spectral power REM sleep spectral amplitude	Higher absolute theta over the left frontal pole region during evening wakefulness, but not during morning wakefulness
					Lower absolute beta spectral amplitude over the primary (*p* < 0.05) and associative (*p* < 0.03) visual areas

van Diessen et al. (53)	*n* = 19; mean age = 10.6 years; sex = 3 F, 16 M	*n* = 19; mean age = 10.1 years; sex = 3 F, 16 M; 7 taking medication	Relaxed, eyes closed	Spectral power	Higher relative gamma power in frontal, parietal, and temporal regions (*p* = 0.002)

Mathewson et al. (54)	*n* = 15; mean age = 18–51 years; sex = 3 F, 12 M; subtypes = autism, Asperger syndrome, PDD-NOS; medication = 8; handedness = 13RH, 2LH	*n* = 16; mean age = 22–47 years; sex = 4 F, 12 M; handedness = 14RH, 2LH	Relaxed eyes opened and eyes closed	Differences in alpha power	Alpha power in each region greater in ASD than in control in the eyes open condition (*p* < 0.05)

Dawson et al. (55)	*n* = 28; mean age = 11 years; sex = 5 F, 23 M; diagnosis = ASD; subtypes = autism, PDD-NOS; peabody picture vocabulary test-revised scores = 39–108; verbal age average = 5.8	*n* = 28; mean age = 11 years; sex = 5 F, 23 M; verbal age average = 16 *n* = 24; mean age = 4.6 years; sex = 2 F, 22 M; verbal age average = 5.7	Relaxed eyes opened	Chronological-age-matched power spectra group comparison	Delta: ASD reduced power in the frontal and temporal regions (*p* < 0.1)
					Theta: ASD reduced power in all three brain regions (*p* < 0.05). Alpha: ASD reduced power in the frontal and temporal regions (*p* < 0.05)
					Beta: no significant differences

Machado et al. (56)	*n* = 11; mean age = 70.3 months; sex = 4 F, 7 M; diagnosis = ASD; subtypes = autism	*n* = 14; mean age = 66.7 months; sex = 5 F, 9 M	Control: relaxed eyes opened; watching a popular cartoon; watching the cartoon without audio	PSD	Decreased PSD in the central region for delta and theta, and in the posterior region for sigma and beta bands, lateralized to the right hemisphere (*p* < 0.05)

Maxwell et al. (57)	*n* = 15; mean age = 15.1 years; sex = 15 M; diagnosis = ASD; subtypes = 14 Asperger syndrome, 1 autism	*n* = 18; mean age = 14.2 years; sex = 18 M	Relaxed eyes opened	Resting gamma power	Decreased gamma power at the right lateral electrodes (*p* = 0.04)

Scope et al. (58)	*n* = 20; mean age = 12 years; sex = 2 F, 18 M; subtypes = 9 autism, 8 Asperger syndrome, 3 PDD-NOS	*n* = 20; mean age = 13 years; sex = 2 F, 18 M	Looking at Gabor patches of different frequencies	Differences in changes in alpha and gamma frequencies of independent components	Induced alpha power of components that were in or near the cingulate gyrus was increased in ASD (*p* < 0.05)

Stroganova et al. (59)	*n* = 40; age range = 3–8 years; sex = 40 M; subtypes = 38 autism, 2 PDD = NOS	*n* = 40; age range = 3–8 years; sex = 40 M	Sustained visual attention	Spectral power	Increase of gamma at the electrode locations distant from the sources of myogenic artifacts (*p* < 0.05)

Stroganova et al. (59)	*n* = 44; age range = 3–8 years; sex = 44 M; diagnosis = ASD; subtypes = 42 autism, 2 PDD-NOS	*n* = 44; age range = 3–8 years; sex = 44 M	Sustained visual attention	Spectral power	Higher amount of prefrontal delta in autism (*p* < 0.05)

Tani et al. (60)	*n* = 20; mean age = 27.2 years; subtype = Asperger syndrome; diagnosis = ASD	*n* = 10; mean age = 26.5 years	Asleep	Spectral power	Non-significant trend toward decreased relative delta power and increased theta power in slow-wave sleep was found in the AS group

Yang et al. (61)	*n* = 5; age range = 16–22 years; sex = 1 F, 4 M; subtype = Asperger syndrome	*n* = 7; age matched	Looking at photographs of familiar faces	Spectral power	Decrease following the stimulus onset in two time-frequency intervals—(1) 150–300 ms in the 1–16 Hz frequency range and (2) 300–650 ms in the 1–8 Hz range (*p* < 0.01)

Tierney et al. (62)	*n* = 168l; diagnosis = ASD	Longitudinal studies: same patients at 6, 9,12, 18,24 months	Resting state	Change over time in spectral power	Across all bands, spectral power was lower in high-risk infants as compared to low-risk infants at 6 months of age (*p* < 0.01)

Sheikhani et al. (51)	*n* = 17; age range = 6–11 years; sex = 4 F, 13 M; diagnosis = ASD; handedness = 1LH, 1 AD, 15 RH	*n* = 11; age range = 6–11 years; sex = 4 F, 7 M	Relaxed eyes opened	Accuracy in differentiating ASD using spectrogram criteria	Alpha frequency band had the best distinction level of 96.4% in relaxed eye-opened condition using spectrogram criteria. ASD had significant lower spectrogram criteria values in left hemisphere (*p* < 0.01), at F3, T3, FP1, F7, C3, Cz, and T5 electrodes (*p* < 0.05)

Lushchekina et al. (63, 64)	*n* = 27; mean age = 5.8 years; diagnosis = ASD; subtypes = autism	*n* = 24; mean age = 6.1 years	Relaxed eyes closed, mental loading (counting, adding and subtracting numbers)	Spectral power in theta and gamma bands	ASD-lower theta spectral power in baseline (*p* = 0.001) and higher gamma spectral power in baseline (*p* = 0.005); cognitive loading presented no changes from baseline in either bands

Lushchekina et al. (63, 64)	*n* = 27; age range = 5–7 years; sex = 27 Mdiagnosis = ASD; subtypes = autism	*n* = 19; age range = 5–7 years; sex = 19 M	Relaxed eyes closed, mental loading (counting, adding and subtracting numbers)	Spectral power in alpha, beta, and gamma bands	The cognitive task led to increases in spectral power in alpha1 and alpha 3 but no changes in alpha 2
					ASD-gamma spectral power higher than control and did not change during the task (*p* < 0.05)

Elhabashy et al. (65)	*n* = 21; age range = 4–12 years; diagnosis = ASD	*n* = 21; age range = 4–12 years	Relaxed eyes open	Absolute and relative spectral power	Increased absolute delta and theta power in ASD especially at the frontal region

*n, number of subjects; F, female; M, male; LH, left-handed; RH, right-handed; AD, ambidextrous; PDD-NOS, pervasive developmental disorder-not otherwise specified; PSD, power spectral density; ASD, autism spectrum disorders*.

Inconsistencies are observed in the spectral band findings, although some generalizations can be inferred. First, significant differences in the alpha band were shown by five studies with relaxed eyes open condition (41, 42, 50, 54, 55). While four studies show a decrease in absolute spectral power in ASD in children of similar ages (41, 42, 50, 55), another showed elevated absolute alpha power in adults (54). The inconsistencies might be attributed to the age differences between participants and differing developmental trajectories in children with ASD. Yang et al. tested participants while they were performing a cognitive task and showed an increase in alpha power compared to non-ASD controls, but insufficient studies calculated similar measures under the same conditions to make robust conclusions (61).

Calculating spectral amplitudes in all frequency bands, Chan et al. also found significantly increased amplitudes in the ASD population. Moreover, using discriminant function analyses, the study finds that beta amplitudes had high specificity (98.1%) and sensitivity (77.8%) in differentiating ASD from non-ASD subjects (70). The most consistent result that could lead to a generalization is an increase in absolute gamma power in ASD compared to non-ASD subjects. Sheikhani et al. tested their participants in a variety of conditions and found statistically significant elevated gamma power, particularly in the relaxed eyes open condition (51). van Diessen et al. confirms the increase in gamma power, particularly in the frontal, parietal, and temporal regions in the relaxed eyes open condition (53). Stroganova et al. replicate this finding, using a visual attention task (59), while Lushchekina et al. supports these findings in two studies involving a cognitive task (64).

As for the theta band, none of the results could be validated due to inconsistencies. While Daoust et al., Tani et al., Elhabashy et al., and Yang et al. show increased power in the theta band in two studies performed during sleep, one during a relaxed eyes open and one involving a cognitive task (52, 60, 61, 65); three studies show a reduction in theta power in relaxed eyes open conditions and during a cognitive task (55, 56, 64). Variations in the participants’ age, as well as small sample sizes might lead to the lack of consistency in these results.

### Information Dynamics

Information dynamics comprises a collection of new methods derived (28) from analysis of non-linear physical systems using new computational methods from the mathematics of complex dynamical systems (71, 72). Of the all studies representing the final literature pool considered in this review, six studies used these interdisciplinary methods to compare ASD patients and non-ASD subjects. These studies may use machine learning algorithms, neural networks, graph theory, or Bayesian methods to find group differences from features computed with non-linear algorithms, such as multiscale entropy or fractal analysis. The challenge with these methods is to determine neurophysiological meaning associated with the measures. All studies reported at least one statistically significant difference in at least one variable measured and very high classification capabilities based on those variables. Of the six studies employing such methods of analysis, only two have a common measure. The other four studies use distinct variables and concepts with the same goal. EEG recordings were performed under relaxed, no task conditions, with eyes either open or closed, or during an object recognition or audio task. The overall patterns in results are presented in Table 5, which show the condition of the EEG recording and the main measure of each study or means of classification.

**Table 5:** Studies using information dynamics.

Paper	Patients characteristics	Controls characteristics	Condition	Measure	Changes in ASD
Bosl et al. (12, 30)	*n* = 46; age range = 6–24 months; diagnosis = HRA	*n* = 33; age range = 6–24 months	Infants’ attention was engaged by the researcher blowing bubbles	mMSE Machine learning classification accuracy	HRA lower mean complexity over all channels; most prominent differences between groups was the change in mMSE between 9 and 12 months Machine learning techniques threshold *p* = 0.05: HRA and control groups classified at age 9 months for boys and girls together and for boys separately with accuracies of 80% and well over 90%, respectively

Eldridge et al. (66)	*n* = 19; age range = 6–10 years; diagnosis = ASD	*n* = 30; age range = 6–10 years	Auditory paradigm (oddball paradigm)	Classification accuracy using robust features, a support vectormachine, logistic regression, and a naive Bayes classifier	Bayesian classification: sensitivity of 79% was achieved for classifying ASD and non-ASD subjects

Gregory and Mandelbaum (67)	*n* = 56; age range = 2–22 years; diagnosis = ASD	*n* = 56; age range = 2–22 years	Relaxed eyes open	Differences in posterior dominant EEG rhythm (PDR) between groups	2-sampled *t*-tests: across the entire pool of participants: significantly different PDR between ASD and non-ASD subjects (*p* = 0.014) ages 2–5.9 years (*n* = 22): significantly different PDR between ASD and non-ASD subjects (*p* = 0.047). ages 6–22 years (*n* = 34): no significant differences in PDR between ASD and non-ASD subjects (*p* > 0.05)

Ahmadlou et al. (68, 69)	*n* = 9; age range = 7–13 years; subtypes = autism	*n* = 9; age range = 7–13 years	Resting state, eyes closed	Discriminative capacity of functional connectivity within and between regions Accuracy of EPNN classification of ASD and non-ASD	One way ANOVAs: Theta band right-temporal-right-temporal; Occipital-Frontal; Parietal-Right-temporal; Occipital-Central (*p* < 0.0005) 95.5% sensitivity with 1.2% variance of classification of ASD and non-ASD subjects

Ahmadlou et al. (69)	*n* = 9; age range = 6–13 years; sex = 2 F, 7 M	*n* = 8; age range = 7–13 years; sex = 2 F, 6 M	Resting state, eyes closed	Discriminative capacity of Fractal Dimensions (FD) in 5 sub-bands Accuracy of Radial Basis Function Neural Network classification of ASD and non-ASD	One way ANOVAsSignificant differences using Katz’s Fractal Dimension: gamma in temporal regions, delta in frontal and central regions (*p* < 0.001) 90% accuracy in the 3 parameter feature space with 0.15% variance

Catarino et al. (13, 39)	*n* = 15; mean age = 31.4 years; diagnosis = ASD	*n* = 15; mean age = 29.4 years	Face and chair detection task	Signal complexity (multiscale entropy)	ASD decreased multiscale entropy over temporo-parietal and occipital regions (*p* = 0.036)

*n, number of subjects; F, female; M, male; HRA, high risk for autism; mMSE, modified multiscale entropy; ANOVAs, analyses of variance; ASD, autism spectrum disorders*.

Multiscale entropy measurements were employed by Catarino et al. and Bosl et al. to measure brain signal complexity in children with autism, infants at HR of developing ASD and non-ASD subjects during a relaxed eyes open condition and an object detection task, respectively. Catarino et al. found significantly decreased multiscale entropy in ASD-diagnosed participants compared to controls, predominantly in temporo–parietal and occipital areas of the brain (13). Bosl et al. found the same results in multiscale entropy, averaged over the entire scalp. The latter found that the greatest differences are observed between 9 and 12 months of age, as multiscale entropy shows an overall different developmental trajectory for infants at HR of developing ASD compared to LR infants. Bosl et al. used classification algorithms to show that children with a HR of developing autism based on family history could be detected with high accuracy at 9–12 months of age, leading to the more important question of whether an autistic neuroelectrophysical phenotype could be detected early in infancy. This supervised learning experiment yielded a sensitivity value of over 80% at 9 months of age, remaining very high (70–90%) until 12 months of age (12).

Elridge et al. used Bayesian methods to perform a similar classification between ASD and typically developing children, between 6 and 10 years old. This study extracted robust features such as variance in time, entropy, or sum of signed differences from the EEG signal and then used logistic regression and a native Bayes classifier to divide the two groups with a 79% accuracy (66). Ahmadlou et al. used a different method of classification based on complexity and chaos theory. Two types of fractal dimensions were used to assess dynamical changes in the brains of ASD children and non-ASD subjects in all frequency sub-bands: Higuchi’s Fractal Dimension and Katz’s Fractal Dimension, which indicate non-integers or fractional dimensions of time series, based on regularity or self-similarity of a time series. After computation of fractal dimensions, the most significant characteristics were extracted using analysis of variance. Finally, a Radial Basis Function Neural Network classifier was used to separate ASD from non-ASD subjects. The accuracy of this classification was 90 % (68).

## Discussion

The aim of this review is to determine the utility of EEG in identifying abnormal activity in brain signal to help in the diagnosis of ASD and for the delineation of its three main subtypes; autism, Asperger syndrome, and PDD-NOS.

Most of the reviewed studies identify differences between ASD and non-ASD subjects regardless of the recording conditions or analysis. However, there is no sufficient evidence to support any of these methods in the diagnosis of ASD. Sources of heterogeneity such as gaps in study designs were identified and recommendation for future research is given.

### Functional Connectivity—Utility, Research Gaps, and Future Directions

Functional connectivity studies showed consistent decreased long-range connectivity in the alpha band and short-range connectivity in the theta band in ASD. However generalizations cannot be inferred yet due to either contradictory results of some studies, or differences in study design. These results only indicate potential reasonable utility of using functional connectivity measures for ASD detection. Methodological challenges involve interpretation of functional connectivity between surface or scalp regions. More than six different methods can be found to define synchronization between time series alone (73). Coherence, correlation, and synchronization can then be used in different ways to determine the strength of connectivity between sensor locations.

It should be noted that EEG recordings usually involve more complex electrode montages and advanced computational capacity for this method of data analysis. These requirements may not necessarily involve the simple, lightweight devices, with high portability that could be operated by non-specialized staff. In addition, the computational challenges required by this type of analysis should not be underestimated. Moreover, since there is little homogeneity in the results obtained using this method, future studies should take into consideration current limitations. Numerous concerns of study design suggest that future research must ensure robust experimental designs and limited reliance on patients with comorbidities, such as epilepsy or subjects taking medication. Epilepsy, one of the most common comorbidities of ASD, is associated with abnormal EEGs and this may complicate the separation of those effects by those caused by ASD (22). Moreover, different types of medication have varied effects on the EEG signal and could be a source of heterogeneity in the results (74). The different means of computing coherence should also be investigated, since different studies yielded contradicting results depending on the analysis method.

In the context of ASD, functional connectivity has not been studied as extensively as spectral analysis. The number of studies identified indicates that coherence may have high utility as a means of accurately detecting ASD. However, the heterogeneity of the coherence results comes partly from a disregard of developmental trajectories of this variable. Previous studies showed that coherence is elevated with age in the high-frequency bands. Children with ASD undergo a slower maturation process and they show generally greater long-range coherence than typical developing children. As coherence increases with age, the values become comparable in adulthood between ASD and non-ASD subjects (75). Thus, age and developmental trajectories must be included in any studies that examine differences between children with ASD and typically developing children.

### Spectral Analysis—Utility, Research Gaps, and Future Directions

Spectral analysis was the most common method of EEG signal interpretation for the detection of ASD identified in this review. The most consistent finding was an increase in absolute gamma power in ASD compared to non-ASD controls. However, inconsistencies between the studies appear due to differences in experimental designs. These may be related to differences in the age of the population studied and failure to fully account for developmental changes.

A major feature of spectral analysis that reinforces its utility for ASD detection is the fact that it does not require elaborate electrode montages, as the analysis can be performed on signals extracted from one single electrode. It is also simple to compute and interpret. However, it is highly reliant on the study condition given that, in essence, it gives a description of the signal in terms of its frequencies, which are dependent on the task performed. For example, greater alpha amplitudes may reflect inhibition of unnecessary activity and better performance on the task, in accordance with the neural efficiency hypothesis (76, 77). Therefore, generalization can only be drawn from studies testing their participants in very similar experimental conditions. Future studies might consider examination of lower frequency bands, such as every 2 or 5 Hz.

Spectral analysis measurements are also dependant on brain maturation and developmental trajectories. For instance, in typically developing children, power distribution tends to shift to higher frequencies with age, with lower frequencies decreasing in relative power (78). Therefore, the age is critical in the interpretation of the results and has to be taken into consideration when comparisons are drawn.

Another important aspect that requires consideration in future studies is whether patients are taking medication. Different types of medicine could influence the EEG signal. For example, many ASD patients receive antiepileptic medication, which shows abnormal brain topography particularly in the gamma band (74).

### Information Dynamics—Utility, Research Gaps, and Future Directions

Information dynamics was used in a limited number of studies in this review. This is due in part to the fact that these methods have been more recently developed in the mathematical community, and are less well known in general as signal analysis tools. Information dynamics computations are also more difficult to perform and fewer ready-to-use software packages are available. The preliminary results, thus, far indicate potential for accurately classifying ASD and non-ASD subjects. However, the limited number of studies must be replicated more widely and with much larger sample sizes before their clinical usefulness can be determined. Conclusions and generalizations on the utility of information dynamics methods cannot yet be drawn as a number of technical difficulties, such as appropriate sample sizes, have to be overcome. A much higher number of studies are required in order to assess and compare these methods with more common spectral power methods used in the field. Further research using measurements such as entropy is encouraged as they interpret the EEG recording as a non-linear signal and bring new perspectives to the field.

### EEG—General Utility

Besides the direct relation between EEG and the neurophysiological features described above, EEG provides a much lower cost, ease of use, as it is portable and can be applied by someone with minimal training, compared to neuroimaging methods. Moreover, EEG data have excellent temporal resolution and measures brain activity directly. This facilitates research as it allows for numerous analyses revolving around direct responses to stimuli, inter-regional connectivity, or topography of brain oscillations. The low cost and ease of use also means that EEG findings have potential clinical application in primary care settings. Therefore, an understanding of qEEG methods and an evaluation of their utility is very important for future research and for the prospective use of EEG in a clinical context. Furthermore, as discussed, developmental trajectories may be more important than single measurements in time for determining functional brain development patterns. The low cost and ease of use of EEG devices is a prerequisite for a brain-based measurement that can be used routinely to monitor brain development.

### Delineation of Subtypes

The second goal of this review was to identify the utility of EEG in the delineation of ASD subtypes. Out of 40 papers, only seven stated the subtype of the ASD group tested. However, testing was not done in comparison between subtypes, which would help delineate them according to one or more characteristics. In addition, none of the studies explores the genetic aspect of neurophysiological subtypes in ASD. Subtypes may be associated with a candidate gene, or complex gene profiles, and, therefore, future studies should consider association between genetic underpinnings and abnormal neurophysiological activity. ASD subtypes also have differences both in their neurological and behavioral manifestations. Moreover, different subtypes may have different developmental trajectories (62). Together, these become sources of heterogeneity in the results. This lack of evidence to help a distinction between different subtypes on the spectrum, represents nothing but strong motivation for further research, with experimental designs that would differentiate, classify, and compare their populations.

Lastly, it is notable that the brain’s electrical activity and indeed overall function necessarily exhibits individual characteristics that vary from subject to subject. Taking this into consideration has a substantial impact on establishing a baseline before any analysis is performed. Even within the broad autism phenotype, there is wide variation in intelligence, social skills, language ability, and other specialized abilities in music, visual arts, and motor development. Deciphering the characteristics that are essential to the autism phenotype within the background of widely varying cofounding functional brain characteristics is a major challenge.

### Conclusion and Recommendations

Overall current EEG signal analysis is not able to identify children with ASD with sufficient sensitivity or specificity to be clinically useful at this time. However, these methods of analysis and their results to date suggest high utility in characterizing the disorder and may be a vital complement to other existing technologies. The use of EEG as a brain development measure may eventually become useful as an indicator that a child requires further evaluation. The current literature supports further research, suggesting different electrophysiological features of high importance and major gaps that can be filled. Bearing in mind that ASD is a neurodevelopmental disorder, diagnosed in childhood, age should be considered when designing the experiment. Moreover, longitudinal studies could reinforce important findings and also delineate developmental stages of ASD. New, advanced methods of analysis should be considered and combined with already established ones in order to fulfill the final goal: early detection of emerging autism, which may open a window for early intervention and prevention.

## Author Contributions

OG developed the protocol, she then followed to perform the literature search, followed by a three-step selection that led to the final pool of articles to review. She performed the data extraction and analysis and wrote the review accordingly. CN and WB supervised the entire process closely, playing a role in the selection of the final literature pool and in the editing of the paper.

## Conflict of Interest Statement

The authors declare that the research was conducted in the absence of any commercial or financial relationships that could be construed as a potential conflict of interest.
